# Thermoelectric Power Generation of TiS_2_/Organic Hybrid Superlattices Below Room Temperature

**DOI:** 10.3390/nano13040781

**Published:** 2023-02-20

**Authors:** Numan Salah, Neazar Baghdadi, Shittu Abdullahi, Ahmed Alshahrie, Kunihito Koumoto

**Affiliations:** 1 Center of Nanotechnology, King Abdulaziz University, Jeddah 21589, Saudi Arabia; 2 K. A. CARE Energy Research and Innovation Center, King Abdulaziz University, Jeddah 21589, Saudi Arabia; 3 Department of Physics, Faculty of Science, King Abdulaziz University, Jeddah 21589, Saudi Arabia; 4 Department of Physics, Faculty of Science, Gombe State University, P.M.B, Gombe 127, Nigeria; 5 Nagoya Industrial Science Research Institute, Nagoya 464-0819, Japan

**Keywords:** TiS_2_/organic superlattice, thermoelectric, TE power generation, ΔT below RT, TE module

## Abstract

Recently, the n-type TiS_2_/organic hybrid superlattice (TOS) was found to have efficient thermoelectric (TE) properties above and near room temperature (RT). However, its TE performance and power generation at the temperature gradient below RT have not yet been reported. In this work, the TE performance and power generation of the TOS above and below RT were investigated. The electrical conductivity (*σ*) and Seebeck coefficient (*S*) were recorded as a function of temperature within the range 233–323 K. The generated power at temperature gradients above (at ΔT = 20 and 40 K) and below (at ΔT = −20 and −40 K) RT was measured. The recorded *σ* decreased by heating the TOS, while |*S|* increased. The resulting power factor recorded ~100 µW/mK^2^ at T = 233 K with a slight increase following heating. The charge carrier density and Hall mobility of the TOS showed opposite trends. The first factor significantly decreased after heating, while the second one increased. The TE-generated power of a single small module made of the TOS at ΔT = 20 and 40 K recorded 10 and 45 nW, respectively. Surprisingly, the generated power below RT is several times higher than that generated above RT. It reached 140 and 350 nW at ΔT = −20 and −40 K, respectively. These remarkable results indicate that TOS might be appropriate for generating TE power in cold environments below RT. Similar TE performances were recorded from both TOS films deposited on solid glass and flexible polymer, indicating TOS pertinence for flexible TE devices.

## 1. Introduction

Global energy demands increase by the day in all sectors of society. This is due to the extensive use of electricity in modern electronic devices and facilities, various mobilities, industries, and infrastructures. However, this has resulted in serious environmental pollution and a climate crisis, particularly due to the use of fossil fuels. As these sources are limited and non-sustainable, it is of great importance to find other energy sources and alternative technologies. One of the promising technologies is converting solar energy or waste heat from various sources into electrical energy using effective thermoelectric (TE) materials. There is a large number of TE materials that have been developed and attempts have been made to enhance/improve these for better TE performance. The first developed TE material was Bi_2_Te_3_ [1], this was then followed by n-type Bi_2_Te_3-x_Se_x_ and p-type Bi_2-x_Sb_x_Te_3_ [2]. Subsequently, a wide variety of TE materials, such as sulfides, selenides, silicides, skutterudites, intermetallic compounds, oxides, organic polymers, and carbon nanomaterials, etc. [3,4,5,6,7,8,9,10,11] have been developed. These were mostly developed as mid- to high-temperature TE materials, some of which include SnSe, SnS_0.91_Se_0.09_, SnTe, GeTe, and Cu_2_Te-based compounds, etc. [12,13,14,15,16,17,18] However, low-temperature TE materials are still rarely developed. Moreover, it is noticed that most of the developed materials were investigated for their power generation at temperature gradients only far above RT.

Thus far, the best low-temperature TE materials are Bi_2_Te_3_-based compounds [19]. However, the generated power using these compounds at low temperatures is rarely reported in the literature. Moreover, improving its intrinsic poor mechanical properties and lowering the content of toxic tellurium is still a big challenge for extensive applications. Afterward, some efforts were made to develop other low-temperature TE materials such as BiSb alloys [20], Ta_4_SiTe_4_ crystal in its one-dimensional form [21], Ce(Ni_1-x_Cu_x_)_2_Si_2_ and CeNi_2_(Si_1-y_Ge_y_)_2_ [22], CoSi and Co_1−*x*_M*_x_*Si [23]. Recent reports on Ag_2_Se showed that this material is a promising low-temperature TE material due to its high *ZT* value, intrinsic semiconductor nature, ultra-high carrier mobility, small density-of-states effective mass, and ultra-low lattice thermal conductivity [24,25]. Mg_3_Bi_2_ [26], Ta_4_SiTe_4_ [27], GeTe-based alloys [28], and silicon thin films [29] were also reported to have moderately good TE performance at low temperatures. Some of these materials seem to be promising; however, the effect of incorporating them with some polymers to produce flexible TE devices is still unknown and their power generation at temperature gradients below RT has not been reported.

For flexible TE materials and devices, highly anisotropic crystals such as CsBi_4_Te_6_ or TiS_2_ might be useful for intercalation with some organic thin layers. Recent reports showed that CsBi_4_Te_6_ has a good TE performance at low temperatures [30]. In addition, TiS_2_ has an anisotropic layered structure with attractive electrical properties. Furthermore, 1T-TiS_2_ has a hexagonal close-packed (hcp) structure, with its layers consisting of covalent Ti-S bonds with a bond length of 2.423 Å. Such layers are bound together by the van der Waals force. Moreover, due to its elegant features such as an attractive structure, lightweight material, cheap chalcogenide, and high electrical conductivity with a semi-metallic behavior [31], TiS_2_ has been employed as a cathode material in rechargeable batteries [32], electrode materials of pseudocapacitors [33], and as a sensor material for uric acid determination [34]. In addition to these applications, the single crystals of TiS_2_ were reported to show good TE performance [35,36,37,38]. It was also involved in nanocomposites [39] for producing flexible TE materials and devices [40,41,42]. Additionally, layered TiS_2_ intercalated with linear alkylamines has recently attracted significant interest as a model compound for flexible n-type thermoelectric applications, showing remarkably high-power factors at RT [43]. The excellent anisotropic property of TiS_2_ led to the novel work on developing a superlattice made of TiS_2_/organic, which seems to be very important from the application point of view. However, the TE performance of the TiS_2_/organic superlattice and its power-generation characteristics below RT have not been reported yet in the literature.

In this work, the n-type TiS_2_/organic hybrid superlattice (TOS) was produced and investigated for its TE performance below RT. Moreover, the power generation at a temperature gradient below RT was measured. The electrical conductivity (σ) and Seebeck coefficient (S) were recorded as a function of temperature within the range 233–323 K. Additionally, the power-generation characteristics at the temperature gradients ΔT = 20 K and 40 K were measured either below or above RT. The charge carrier density and Hall mobility of TOS were measured at 233–323 K. The generated maximum power of a small module made of TOS (formed on a glass substrate) at ΔT = 20 and 40 K above RT were compared with those generated below RT. The obtained results are discussed in detail and showed the significant potential of this material to be used for generating TE power in cold environments.

## 2. Materials and Methods

Synthesis of the TiS_2_/organic superlattice thin film was conducted in this work, similar to that reported by Tian et al. [40]. Commercially available TiS_2_ powder of high purity (99.9%) with a particle size of around 200 mesh was obtained from Sigma Aldrich, Bengaluru, India. While hexylamine, HA (99.0%,) and N, N-dimethylformamide, DMF (99.0%) were supplied from Sigma Aldrich, Darmstadt, Germany. Initially, TiS_2_ powder was mixed and ground with hexylamine (HA) in an agate mortar with a molar ratio of 1:4, and this resulted in a metallic brown powder. This resultant powder was further exfoliated into nanosheets by adding DMF as a highly polar solvent. A volume ~20 times that of the HA was added. This addition was followed by sonication. The obtained colloidal solution was then centrifuged to remove the unintercalated TiS2 and thick flakes. The exfoliated nanosheets were added into a petri dish containing a glass slide to make thin films on. The petri dish was inserted inside a vacuum furnace at 60 °C for drying. The obtained films formed on the glass slide had a thickness of 40 µm. The film was further annealed at 403 K for 5 h. A polymer substrate was also used to produce a flexible TE material. This polymer is a polyethylene terephthalate (PET) sheet of a thickness of around 20 µm. It was supplied by Fuji Film Holdings, Japan.

The films were characterized using several techniques, such as scanning electron microscopy (SEM) (JSM-7500F, JEOL, Tokoyo, Japan), transmission electron microscopy (TEM) (JEM 2100F, JEOL), and X-ray diffraction system (XRD) (ULTIMA IV, Rigaku, Japan). The electrical conductivity, Seebeck coefficient, and thermal conductivity in the in-plane directions were recorded for the prepared film as a function of temperature. An LSR-3 Linseis-Seebeck coefficient and electric resistivity system manufactured by Linseis, Germany was used in a helium atmosphere to measure the films’ resistivities and Seebeck coefficients. The heating rate and temperature gradient between the hot and cold sides were fixed at 5 K/min and 50 K, respectively. The charge carrier concentration and Hall mobility were measured using the HCS 10 system, Linseis. A single leg of the TE module made of a film of dimensions: thickness × length × width = 0.03 × 8 × 8 mm was constructed. The in-plane thermal conductivity of the fabricated TOS was determined using the laser flash method in LFA-1000 (Linseis, Selb, Germany). The measurements were performed using a sample holder made of graphite supplied by Linseis. The measurement was conducted in a vacuum atmosphere and the heating rate was set at 10 K/min. The TE leg was perpendicularly fixed using a stand made of a ceramic plate, while aluminum electrodes were used to attach both sides of the TE leg to the measurement systems. To control the temperature gradient between two sides of the leg, a hot plate, solid ice, cold water, and liquid nitrogen were employed to generate the temperature differences (a table and figure describing this arrangement are included in the following sections). An infrared temperature gun to measure the temperature at the edge of the leg was used. The power-generation characteristics of this single leg were investigated at different temperatures under real-time conditions in the air.

## 3. Results and Discussion

**Figure 1:**
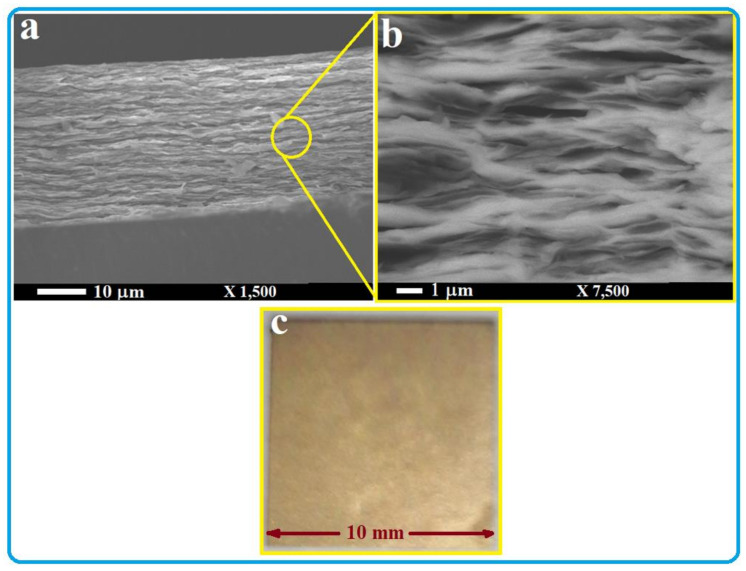
SEM images (**a**,**b**) of the synthesized film of TiS_2_/organic superlattice. Photograph image of a top view of the film formed on a 10 × 10 mm glass substrate is also shown (**c**).

**Figure 2:**
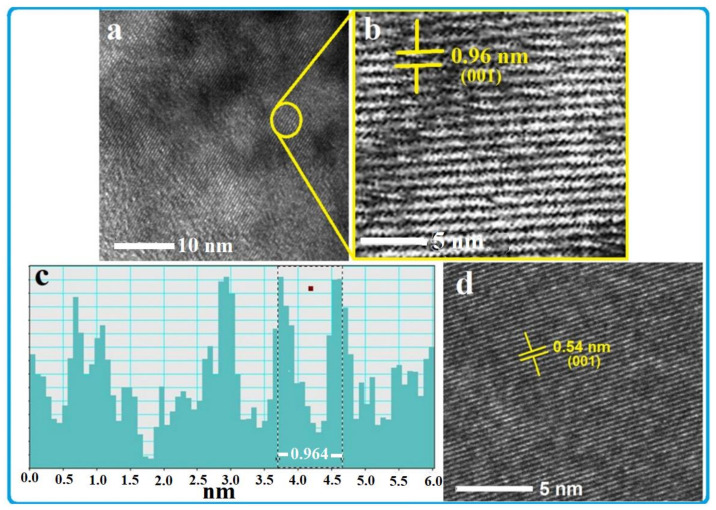
HRTEM images (**a**,**b**) of TiS_2_/organic superlattice and line scan (**c**) of the HRTEM image showing a d-spacing of ~9.64 Å. The HRTEM image presented in (**d**) is of a TiS_2_ single crystal.

The surface morphology and microstructure of the produced TiS_2_/organic superlattice (TOS) film on a glass substrate were investigated by both SEM and TEM techniques and the obtained result is presented in Figure 1 and Figure 2. The SEM images shown in Figure 1a,b clearly display the aligned layers/flakes of TOS with a thickness of less than 100 nm. Some gaps can be seen between these layers. These layers/flakes of TOS seem not to be completely stacked with each other, at least in those which are close to the edges. Nevertheless, this structure and these layers are similar to those reported previously [40,41,42,43,44,45]. The film color is also like those reported in the literature, as shown in the photographed image presented in Figure 1c, which shows a top view of the film formed on a 10 × 10 mm glass substrate. HRTEM images of a TOS film were also recorded at two different magnifications and are presented in Figure 2a,b. These images show the atomic and sub-atomic details of a single layer/flake of TOS, which firmly indicate that a nearly perfect stacking of a TiS2/organic layer-by-layer structure was achieved. The line scan of the HRTEM image is presented in Figure 2c, which shows the d spacing of the corresponding (001) plane is ~9.64 Å. This value is much larger than that of a TiS_2_ single crystal, ~5.4 Å [38], as shown in Figure 2d, which also confirms that a nearly perfect superlattice composed of TiS_2_/organic alternate stacking layers was formed [44].

**Figure 3:**
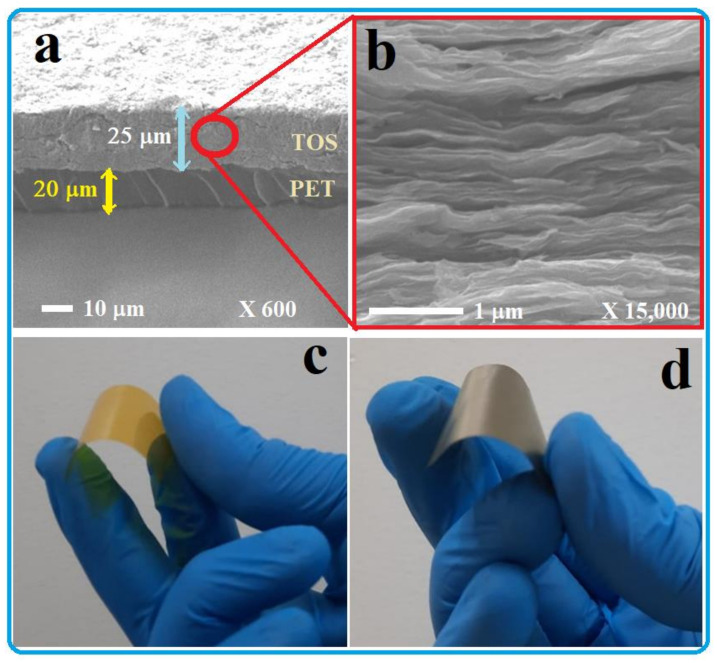
(**a**) SEM image of the TiS_2_/organic superlattice deposited on a PET substrate. (**b**) High-magnification SEM image of the TOS deposited on the PET substrate. Photographs of the PET film (**c**) without TOS and (**d**) with deposited TOS.

As mentioned above in the experimental section, a polymer substrate was also used to produce a flexible TE material. This polymer was chosen to be a polyethylene terephthalate (PET) sheet of a thickness of around 20 µm (Figure 3). In Figure 3a, a SEM image of the TOS deposited on the PET substrate is shown. The thickness of the deposited TOS is around 25 µm. The layers shown in Figure 3b are like those deposited on a glass substrate (Figure 1b). The photographs of the PET film without and with TOS are shown in Figure 3c,d, respectively. The deposited TOS film was found to be very stable even with bending the film several times. No crack formation or significant effect on TE performance was observed. Its TE performance and power generation are similar to those of the film deposited on a solid-glass substrate (shown in the next sections). This is quite important to fabricate TE materials for different systems and flexible devices.

**Figure 4:**
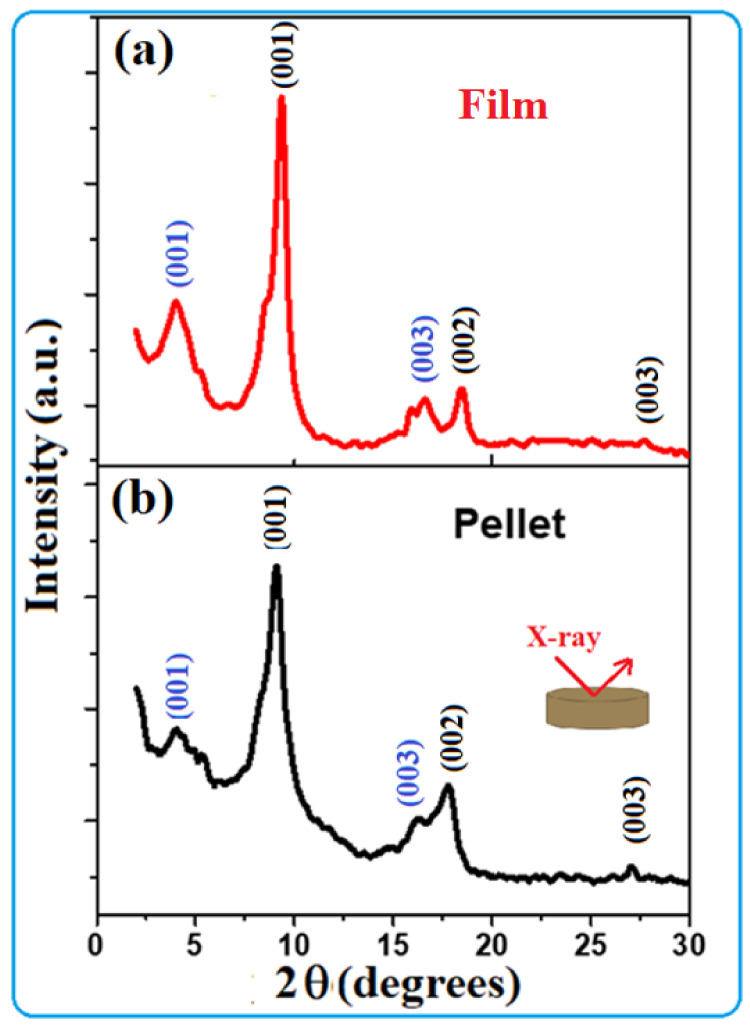
XRD patterns of TOS formed as (**a**) a film on a glass substrate, and (**b**) a pressed compact of the collected powder formed on the bottom of the petri dish without using a substrate.

In addition to the above investigations, which described the surface morphology and microstructure of the TOS (Figure 1 and Figure 2), the XRD pattern of the film formed on a glass substrate as well as that of a pressed compact of collected powder (collected without using any substrate) was recorded. These measurements were recorded after annealing these samples at 403 K for 5 h. The obtained results are presented in Figure 4a,b. The XRD pattern of the TOS film displays several peaks. These peaks were reported to be of two intercalated phases due to the different arrangements of organic molecules in the van der Waals gap of TiS_2_ [40]. The hkl values were labeled in two different colors: blue for phase one and black for the second phase. These two phases were reported to have different lattice parameters c, with values of 17.1 Å and 9.92 Å. In the present samples, the major phase present in both the film and the pellet (Figure 3a,b) is that of the lattice parameter 9.92 Å. The HRTEM image presented in Figure 2b shows a d spacing of 9.64 Å, indicating that this phase is the major one present here. Nevertheless, these results are similar to those produced and evaluated previously [40].

**Figure 5:**
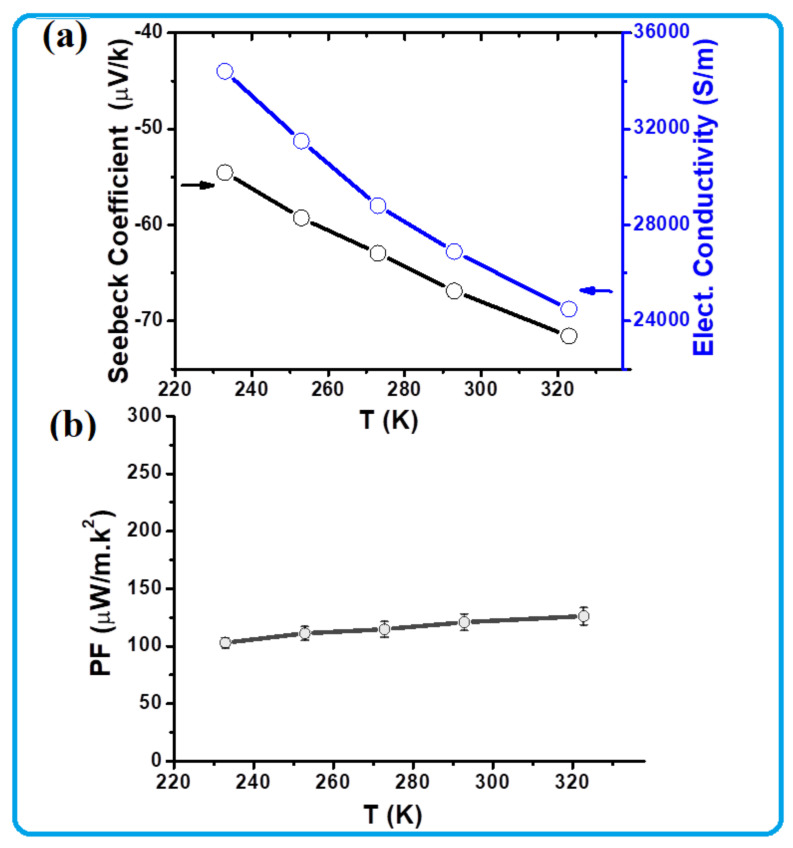
TE performance of the TOS film as a function of temperature.

Seebeck coefficient at 233 K was recorded as −55 µV/K. This value increased to approximately −72 µV/K by heating the film to 323 K. The obtained values are all negative, indicating that this semiconductor has n-type carriers, which is similar to that of TiS_2_ reported by many workers [35,36,37,38]. The calculated power factor, *PF,* is shown in Figure 5b. The obtained value at 233 K is ~104 µW/mK^2^. This value is found to slightly increase with heating. It recorded around 120 µW/mK^2^ at 323 K. This behavior proved to be of almost a degenerate semiconductor, confirming its suitability as an n-type TE material.

**Figure 6:**
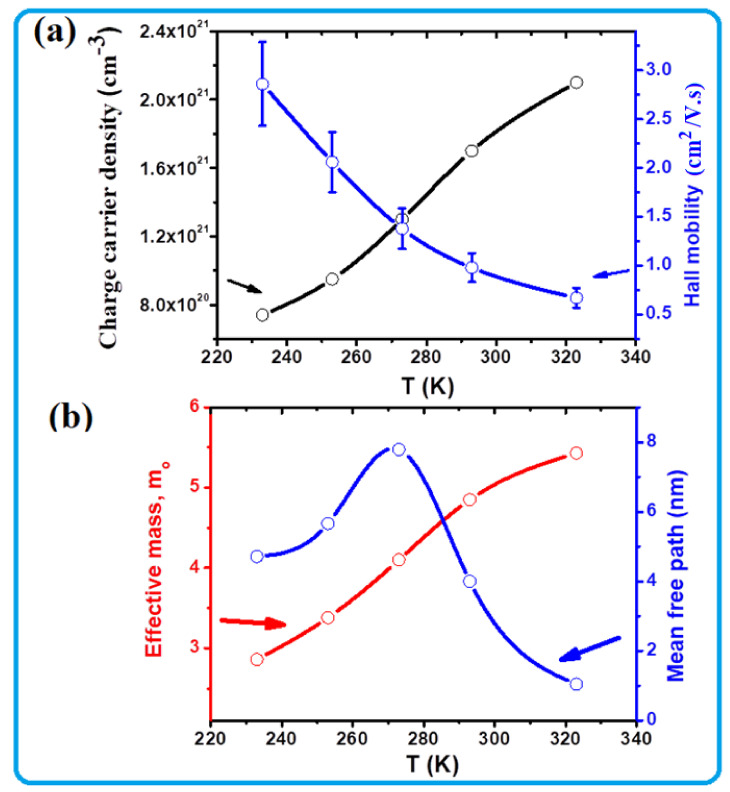
(**a**) Charge carrier density, Hall mobility, and (**b**) effective mass and mean free path of the free charge carriers in TOS as a function of temperature.

The charge carrier density, Hall mobility, charge carrier effective mass, and charge carrier mean free path of the TOS film were also recorded as a function of temperature and the obtained results are presented in Figure 6. It is clear from Figure 5a that the charge carrier density and Hall mobility curves have opposite trends against temperature. The recorded carrier density at 233 K is ~0.75 × 10^21^ cm^−3^, but it increased by a factor of three with heating up to 323 K. The Hall mobility is ~2.8 ± 0.5 cm^2^/Vs at 233 K but decreased to approximately 0.65 ± 0.11 cm^2^/Vs by heating up to 323 K. The obtained charge carrier density and mobility values in this study are close to those reported in the literature [40,46]. However, the observed behavior of increasing the charge concentration by heating might be due to the generation of more carriers and their participation in the electrical conduction, while the decrease in their mobility might have occurred as a result of lattice expansion, which would affect the available carrier channels.

To further investigate the electrical conductivity, the effective mass (m*) and mean free path of the free charge carriers in TOS as a function of temperature were calculated and plotted in Figure 6b. It is clear from the above result that the electrical conductivity (Figure 5a) of the TOS film seems to be of a degenerate semiconductor; therefore, the effective mass can be obtained from the Seebeck coefficient, *S,* and the carrier concentration, *n*, according to Pisarenko’s relationship [47]:

(1) $$S=\frac{8\pi^{2}k_{B}{}^{2}}{3eh^{2}}m^{*}T{\left(\frac{\pi }{3n}\right)}^{2/3}$$

where *k_B_* is Boltzmann constant = 1.38 × 10^−23^ m^2^ kgs^−2^ K^−1^, *h* is Planck’s constant= 6.63 × 10^−34^ m^2^kg/s, and *e* = the electron charge = 1.6 × 10^−19^ Coulomb.

The electron mean free path *l* was deduced from the Fermi velocity *v*_F_ and the scattering time (or relaxation time) *τ* [46]:

(2) $$l=\nu_{F}\tau$$

The relaxation time (*τ*) can be calculated using the following equation:

(3) $$\tau =\frac{\sigma m^{*}}{ne^{2}}$$

The Fermi velocity at the Fermi surface was calculated using the following equation [48]:

(4) $$\nu_{F}=\frac{\hbar k_{F}}{m_{0}}=\frac{\hbar }{m_{0}}{\left(3\pi^{2}n\right)}^{\frac{1}{3}}$$

where ħ = reduced Planck constant, $m_{0}\;\;$= electron mass, and $k_{F}\;\;$= the Fermi wavevector.

The effective mass values were found to increase with heating from around 2.8 *m*_0_ at 233 K to approximately 5.5 *m*_0_ at 323 K (Figure 6b). This high value and its increase with increasing temperature is the main reason for the obtained Seebeck coefficient, whose absolute value also increased with heating. In the case of the mean free path, the maximum value, 8 nm, was detected at ~273 K. Below 273 K, the mean free path reduced to 5 nm, while above 273 K, this value significantly decreased, to 1 nm at 323 K. The mean free path of 8 nm seems to be close to the interlayer distance of TOS, but by changing temperature, the interlayer distance might have decreased due to either layer expansion or shrinkage. However, it is difficult to model the relationship between the carrier mean free path and lattice distortion; thus, this phenomenon should be subject to further investigation.

The thermal conductivity ($\kappa_{\mathrm{total}}$) of a material is the sum of electronic thermal conductivity ($\kappa_{e}$) and lattice (phonon) thermal conductivity ($\kappa_{p}$). The value of $\kappa_{e}\;$ can be obtained using the following Wiedemann–Franz law [49] after measuring the electrical conductivity.

(5) $$L=\frac{\kappa_{e}}{\sigma T}=\frac{\pi^{2}k_{B}^{2}}{3e^{2}}=2\times 10^{-8}{\;W\Omega K}^{-2}$$

where L is the Lorenz number, $k_{B}\;$ is Boltzmann’s constant, and e is the electron charge. The values of the recorded in-plane ${\;\kappa }_{\mathrm{total}}$,${\;\kappa }_{p}$ and ${\;\kappa }_{e}$ of the present TOS are presented as a function of temperature in Table 1. They were recorded within the range 298–363 K. At 298 K, the $\kappa_{\mathrm{total}}$ of the TOS was equal to 0.76 W/mK. When the temperature was increased to 363 K, this value increased to around 0.97 W/mK. The $\kappa_{\mathrm{total}}$ seems to be temperature-dependent within this temperature range. Nevertheless, these values are comparable to those published in the literature [41]. As the temperature increases, the TOS layers may expand, resulting in close contact between them. Consequently, it may reduce phonon scattering sites and enhance phonon transport, which would lead to increased thermal conductivity, κ_p_. The κ_e_ of the TOS is small compared to its $\kappa_{\mathrm{total}}$, while the κ_p_ is closer to its $\kappa_{\mathrm{total}}$, as displayed in Table 2. This indicates that the phonons are the major heat carriers in the TOS. At temperatures below 298 K the values of $\kappa_{\mathrm{total}}$ might be much smaller; this will be considered in our future work. The assessment of the figure of merit (*ZT*) as a function of temperature within the range 298–363 K of the present TOS is shown in Table 1. *ZT* is almost independent of temperature within this temperature range, while *PF* increased with increasing temperature.

**Figure 7:**
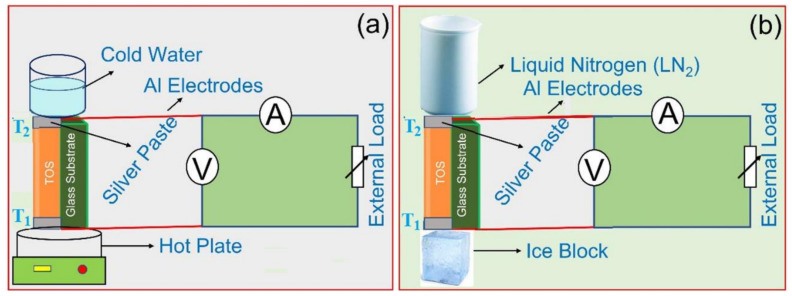
A schematic diagram of the TE device structure and the user setup to measure the power-generation characteristics of a single-leg module of TOS film (**a**) above (ΔT = 20 K and 40 K) and (**b**) below RT (ΔT = −20 K and −40 K).

**Figure 8:**
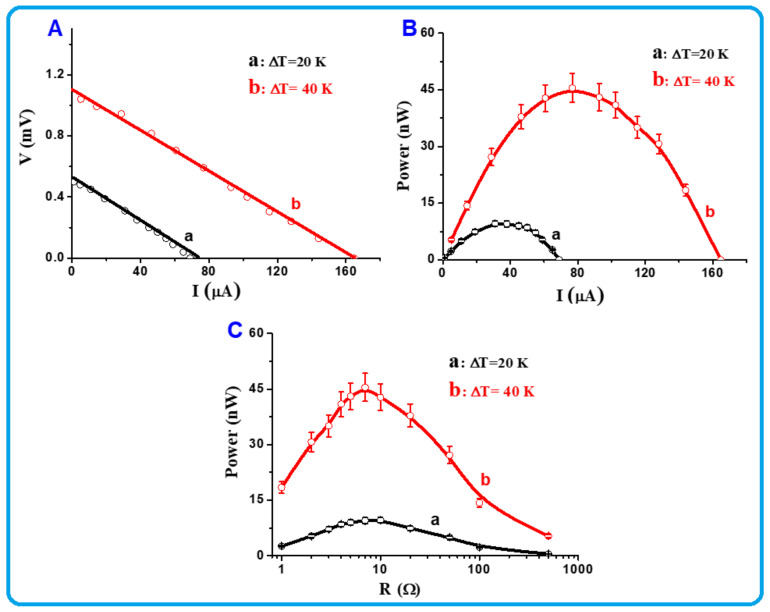
(**A**–**C**) Above/near RT thermoelectric power characteristics of TOS single leg module at ΔT = 20 K and 40 K (for ΔT = 20 K; T_1_ = 283 K and T_2_ = 303 K, for ΔT = 40 K; T_1_ = 293 K and T_2_ = 333 K).

**Figure 9:**
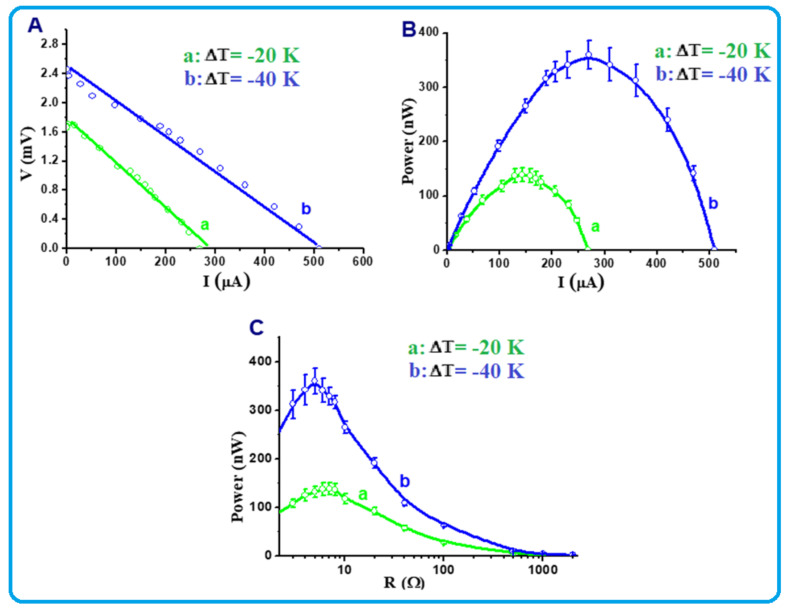
(**A**–**C**) Below RT thermoelectric power characteristics of the TOS single-leg module at ΔT = −20 K and −40 K (for ΔT = −20 K: T_1_ = 273 K and T_2_ = 253 K, for ΔT = −40 K: T_1_ = 273 K and T_2_ = 233 K).

The TE power characteristics of a single-leg module of the TOS film above/near RT at two different temperature differences, ΔT, above and below RT were investigated. As mentioned above, a thin film formed on a glass substrate with thickness = 25 µm, length = 8 mm, and width = 8 mm was used. Table 2 summarizes the selected temperatures and temperature gradients, ΔT was used to measure the power generation produced from this single-leg module. Figure 7 shows a schematic diagram of the single-leg module and the used setup to measure the power generation of this TE device above (Figure 7a) and below (Figure 7b) RT. The obtained results were plotted and are presented in Figure 8 and Figure 9, respectively. When ΔT was fixed at 20 K and 40 K above RT, the recorded short-circuit currents were found to be approximately 75 and 165 µA, while the open-circuit voltages reached 0.5 and 1.1 mV, respectively (Figure 8A). The corresponding power values were plotted as a function of current and are shown in Figure 8B. At ΔT = 20 K, the maximum power was recorded around 10 nW, while at ΔT = 40 K, the maximum power reached 45 nW. These values are reasonable for a single leg made of a small foil [40]. The obtained power values were plotted as a function of load and are shown in Figure 8C. The curves in this figure clearly show that the maximum power values were obtained at an external load of approximately 7 Ω for both ΔT = 20 K and 40 K. This indicated that the internal resistance of the used film is close to 7 Ω. This value is low, which is consistent with the value of the electrical conductivity of the present TOS film.

The generated power below RT for the single-leg module made of the TOS film was also measured and the obtained results are shown in Figure 9. Two temperature gradients, ΔT, were selected, −20 K and −40 K, as shown in Table 2. Similarly, when ΔT was fixed at −20 K and −40 K below RT, the recorded short-circuit currents were found to be approximately 280 and 520 µA, while the open-circuit voltages reached 1.7 and 2.5 mV, respectively (Figure 9A). The corresponding power values were plotted as a function of current and are shown in Figure 9B. At ΔT = −20 K, the maximum power recorded was around 140 nW, while at ΔT = −40 K, the maximum power reached 350 nW. Surprisingly, these values were found to be several times higher than those obtained at temperature gradients above RT (Figure 8B). The obtained power values were also plotted as a function of load and the obtained results are shown in Figure 9C. The curves in this figure clearly show that the maximum power values were obtained at an external load of approximately 4–5 Ω for both ΔT = −20 K and −40 K. This indicated that the internal resistance of the used foil is close to this range, e.g., 4–5 Ω. This value is lower than that obtained when the value of ΔT was fixed above RT (Figure 8C). Although the achieved power density of this single small module (area = 64 mm^2^) was found to be 5.47 mW/m^2^ at ΔT = −40 K, the obtained results can still be further enhanced by changing the polar organic molecules used in the superlattice formation, as reported recently by another research group [50].

The new remarkable finding on TOS in this work is the generation of highly improved TE power below RT, which is several times higher than that generated above/near RT at the same ΔT values. Similarly, here, below RT, the generated current is very high compared to the generated voltage, suggesting that this kind of TE module is suitable as a current source for some devices requiring higher currents. This might be useful in cold-environment regions, particularly in the winter season. The reason behind this improvement below RT might be related to the enhancement of both electrical conductivity and the Seebeck coefficient occurring below RT, as shown in Figure 5a. The reduction in thermal conductivity (hopefully below RT), as shown in Table 2, would keep a comparatively high *ZT*, giving credit for energy conversion efficiency below RT.

The present remarkable findings would firmly suggest that the TOS films might be useful to generate TE power in cold environments. Moreover, the TOS film can be formed on a solid-glass substrate or a flexible-polymer substrate (Figure 3) and both can show almost the same TE performance. This feature can extend the TOS application as a promising heat-driven power source for a wide range of flexible/wearable electronic systems.

## 4. Conclusions

In this work, the n-type TiS_2_/organic hybrid superlattice (TOS) was found to have efficient TE properties below RT. In particular, a single-leg module made up of a TOS film showed remarkable power-generation characteristics. The generated TE power below RT was found to be more than eight times higher than those generated above RT. It was recorded as 140 and 350 nW at ΔT = −20 K and −40 K, respectively. This might be related to the enhancement of both electrical conductivity and the Seebeck coefficient, while rather high *ZT* was maintained below RT due to effectively lowered thermal conductivity; however, this phenomenon needs to be further investigated in future work. From the application point of view, this finding would suggest that TE TOS devices might be useful for small-scale TE power generation in cold environments, for which largely extended modules might be designed and fixed to house windows, for instance, to generate TE power from the temperature difference between inside and outside.

## Figures and Tables

**Table 1:** The in-plane total thermal conductivity, 𝜅_𝑡𝑜𝑡𝑎𝑙_, phonon thermal conductivity, 𝜅_𝑝_, electronic thermal conductivity, 𝜅_𝑒_, and figure of merit, *ZT* of the TiS_2_/organic superlattice film as a function of temperature within the range 298–363 K. The values of the density; specific heat capacity, *C*; thermal diffusivity, *D*; electrical conductivity, *σ*; and power factor, *PF* of the TOS film are also shown in this temperature range.

*T* (K)	Density (g/cm^3^)	*C* (J/gK)	*D* (cm^2^/s)	$\kappa_{total}\;(W/\mathrm{mK})$	$\kappa_{p}\;\;(W/\mathrm{mK})$	$\kappa_{e}\;\;(W/\mathrm{mK})$	*σ* (S/m)	*PF* (µW/mK^2^)	*ZT*
298	0.96	0.582	0.0136	0.76	0.60	0.160	27,000	120	0.047
323		0.585	0.0145	0.81	0.65	0.156	24,200	124	0.049
348		0.601	0.0156	0.94	0.792	0.148	21,300	127	0.047
363		0.619	0.0164	0.97	0.840	0.131	18,100	129	0.048

**Table 2:** The selected temperatures and temperature gradients are used to measure the power-generation characteristics of a single-leg module of TOS film above/near and below RT.

	T_1_ (K)	T_2_ (K)	ΔT = T_2_ − T_1_ (K)	Remarks
Above/near RT	283	303	20	A heater was used at T_2_, cold water at T_1_
	293	333	40	A heater was used at T_2_, RT air at T_1_
Below RT	273	253	−20	Liquid nitrogen with a thin ceramic plate was used at T_2_, solid ice at T_1_
	273	233	−40	Liquid nitrogen was used at T_2_, solid ice at T_1_

## Data Availability

The data supporting the findings of this study are available upon request to nsalah@kau.edu.sa.
